# Exceptionally High Blocking Temperature of 17 K in a Surface‐Supported Molecular Magnet

**DOI:** 10.1002/adma.202102844

**Published:** 2021-08-15

**Authors:** Fabian Paschke, Tobias Birk, Vivien Enenkel, Fupin Liu, Vladyslav Romankov, Jan Dreiser, Alexey A. Popov, Mikhail Fonin

**Affiliations:** ^1^ Department of Physics University of Konstanz 78457 Konstanz Germany; ^2^ Leibniz Institute for Solid State and Materials Research (IFW Dresden) 01069 Dresden Germany; ^3^ Swiss Light Source Paul Scherrer Institute Villigen 5232 Switzerland

**Keywords:** dimetallofullerenes, electrospray deposition, graphene, relaxation, single‐molecule magnets

## Abstract

Single‐molecule magnets (SMMs) are among the most promising building blocks for future magnetic data storage or quantum computing applications, owing to magnetic bistability and long magnetic relaxation times. The practical device integration requires realization of 2D surface assemblies of SMMs, where each magnetic unit shows magnetic relaxation being sufficiently slow at application‐relevant temperatures. Using X‐ray absorption spectroscopy and X‐ray magnetic circular dichroism, it is shown that sub‐monolayers of Dy_2_@C_80_(CH_2_Ph) dimetallofullerenes prepared on graphene by electrospray deposition exhibit magnetic behavior fully comparable to that of the bulk. Magnetic hysteresis and relaxation time measurements show that the magnetic moment remains stable for 100 s at 17 K, marking the blocking temperature *T*_B(100)_, being not only in excellent agreement with that of the bulk sample but also representing by far the highest one detected for a surface‐supported single‐molecule magnet. The reported findings give a boost to the efforts to stabilize and address the spin degree of freedom in molecular magnets aiming at the realization of SMM‐based spintronic units.

## Graphical abstract

Sub‐monolayers of Dy_2_@C_80_(CH_2_Ph) dimetallofullerene single‐molecule magnets are prepared on a graphene monolayer by electrospray deposition. Magnetic hysteresis and relaxation time measurements show that the magnetic moment remains stable for 100 s at 17 K, marking the blocking temperature *T*_B(100)_, being not only in excellent agreement with that of the bulk but also representing by far the highest one detected for a surface‐supported single‐molecule magnet.

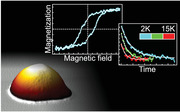

## 1 Introduction

Single‐molecule magnets (SMMs) belong to the smallest units wherein magnetic moments can remain intrinsically stable on time scales required for possible technological applications.^[^ ^1^, ^2^, ^3^ ^]^ Intense research in the field of nanomagnetism within the last decade has further boosted technologically relevant performance indicators, resulting in SMMs magnetically stable above liquid‐nitrogen temperature^[^ ^3^ ^]^ or single atoms exhibiting extremely long magnetic relaxation times.^[^ ^4^, ^5^, ^6^ ^]^ In particular, systems based on late lanthanide family elements, like Dy and Tb, have been largely in focus, including single‐molecule,^[^ ^2^, ^3^ ^]^ single‐atom,^[^ ^4^, ^5^ ^]^ or single‐chain magnets.^[^ ^7^, ^8^ ^]^ Adsorption of SMMs on surfaces allows to study individual molecular units, as well as to realize transport schemes essential for the implementation of SMMs in molecular‐scale spintronics or quantum computing devices.^[^ ^9^, ^10^, ^11^, ^12^, ^13^, ^14^, ^15^, ^16^, ^17^ ^]^ However the transition from bulk to surface‐supported systems often goes along with a substantial change or even loss of SMM properties, that is, magnetic moment, magnetic anisotropy, or magnetization behavior.^[^ ^18^, ^19^, ^20^, ^21^ ^]^ On metallic surfaces, the interaction of the magnetic moments with the surface is rather strong, which is evidenced by the observation of the Kondo effect.^[^ ^22^, ^23^ ^]^ Thus, benchmark measurements during the last years demonstrating magnetic bistability of surface‐adsorbed SMMs have been reported on substrates, where molecules are electronically weakly coupled to – TbPc_2_ on HOPG,^[^ ^24^ ^]^ on MgO/Ag(100)^[^ ^25^ ^]^ and on graphene/SiC,^[^ ^26^ ^]^ pushing the blocking temperature (*T*_B_) limit up to 9 K. On the other hand, DySc_2_N@C_80_ monolayers on Au(111)^[^ ^27^ ^]^ recently showed a hysteresis opening at temperatures up to 10 K. In this sense, lanthanide ions encaged in C_80_ molecules reportedly outperform most SMMs by their combination of chemical robustness with slow magnetic relaxation.^[^ ^27^, ^28^, ^29^, ^30^, ^31^ ^]^ To further push the magnetic lifetime in the monolayer regime two important criteria have to be fulfilled: the first requirement is to synthesize SMM compounds showing intrinsically high *T*_B_ in the bulk. The second requires implementation of the appropriate methods for molecular deposition on substrates, which provide sufficient decoupling of the SMM from the surface.

In this work we provide experimental evidence on outstanding slow magnetic relaxation in Dy_2_@C_80_(CH_2_Ph) sub‐monolayers on a graphene/Ir(111) surface. The Dy_2_@C_80_(CH_2_Ph) molecules deposited by the electrospray deposition method are organized into islands as shown by low‐temperature scanning tunneling microscopy (STM) imaging. We explore their magnetic properties by means of X‐ray absorption spectroscopy (XAS) and X‐ray magnetic circular dichroism (XMCD) measurements. The analysis of the magnetic relaxation behavior of Dy_2_@C_80_(CH_2_Ph) adsorbed on graphene/Ir(111) yields a blocking temperature value not only similar to that of the bulk, but also representing the highest reported one for a molecular magnet on a surface.^[^ ^25^, ^26^, ^27^ ^]^

## 2 Results and Discussion

**Figure 1:**
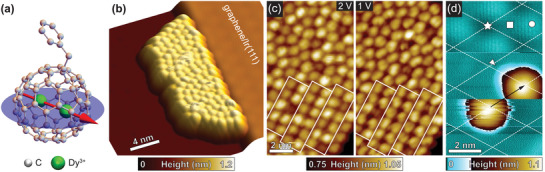
Surface‐assembly of Dy_2_@C_80_(CH_2_Ph) on graphene/Ir(111). a) Molecular structure of Dy_2_@C_80_(CH_2_Ph) as obtained from single‐crystal X‐ray diffraction experiments.^[^ ^28^ ^]^ The blue plane denotes the rotational degree of freedom of the [Dy^3+^–*e*–Dy^3+^] unit, which exhibits a magnetic easy axis that is indicated as the red arrow. H atoms at the benzyl group are omitted for clarity. b) 3D representation of an STM topography showing an ordered Dy_2_@C_80_(CH_2_Ph) molecular island formed at a step of the graphene/Ir(111) surface. Measurement parameters: *U* = +1.5 V, *I* = 10 pA, *T* = 3 K. c) High‐resolution STM images of the molecular arrangement within an island recorded at different bias voltages. The white rectangles denote the formation of a stripy molecular pattern. Measurement parameters: *I* = 10 pA, *T* = 2.7 K. d) STM topographic image of an isolated molecule that is displaced by the STM tip during scanning from bottom to top. The position change is indicated by the black arrow. The dashed lines indicate the graphene moiré superlattice with different regions accordingly: fcc (star), hexagonal close packed (hcp) (square), and atop (circle). The white triangle denotes the protruding benzyl group. The upper third of the image: *U*_gr_ = +0.1 V, *I*_gr_ = 0.1 nA, *T* = 2.9 K; the rest of the image: *U*_mol_ = +1.5 V, *I*_mol_ = 10 pA.

The outstanding bulk magnetic properties of Dy_2_@C_80_(CH_2_Ph) dimetallofullerene (see **Figure** **1**a) stem from a single‐electron bond connecting the two rare earth atoms within the C_80_ cage. Dy^3+^ ions with their spin states of *J*_z_ = 15/2 are ferromagnetically coupled in the ground state. Recent studies^[^ ^28^ ^]^ showed that the easy magnetization axis is aligned along the [Dy^3+^–*e*–Dy^3+^] moiety, which is oriented roughly perpendicular to the C—C bond connecting the CH_2_Ph side group to the C_80_ cage. Necessary to stabilize the electronic structure of the molecular core, the benzyl addend represents one of the smallest linker group, which still allows to assemble and controllably address these SMMs in possible applications. On the other hand, it reduces the stability of the compound hampering thermal evaporation and thus requires an alternative deposition route to study their properties on a surface under clean environment.

Here, sub‐monolayers of Dy_2_@C_80_(CH_2_Ph) molecules were deposited on a graphene/Ir(111) substrate by employing the electrospray deposition (ESD) technique^[^ ^32^, ^33^, ^34^ ^]^ (see Section 4 for further details). Our STM measurements show that the majority of molecules constitute ordered islands with only small amount of isolated molecules lying on the surface. Figure 1b shows an STM topographic image of a molecular island with an apparent height of 0.93 ± 0.05 nm. Depending on the bias voltage, Dy_2_@C_80_(CH_2_Ph) molecules show spherical or slightly elliptical shape with mean diameter of about 1 nm as can be seen in Figure 1c. We assign the asymmetry of appearances to be mainly reflecting the position of the CH_2_Ph side group, and thus giving a reasonable guess for the respective molecule's orientation. At a bias voltage of 1 V, both elongated shapes as well as double‐lobe structures can be distinguished, indicating different spatial orientations of the molecules with respect to the side group, when embedded in close‐packed islands. At a higher bias voltage of 2 V, the different rotational configurations are reflected by a small variation of molecular apparent height in the range of ±50 pm. This observation yields a pronounced scatter for the direction of the magnetic anisotropy axis of Dy_2_@C_80_(CH_2_Ph) on the graphene surface.^[^ ^27^ ^]^ Furthermore, due to the presence of the side group, the molecules tend to order in a stripy pattern within the islands. Whereas these findings reflect the situation of the majority of molecules in the sample, the molecule‐substrate interaction can be better evaluated by imaging a rarely found isolated SMM on the surface. Figure 1d shows the tip‐induced displacement of a single molecule during scanning at moderate tunneling parameters. Albeit the weak pinning, we observe the favored adsorption site for Dy_2_@C_80_(CH_2_Ph) to be the face‐centered‐cubic (fcc) region of the graphene moiré superlattice. Furthermore a small protrusion to the side indicates the side group lying flat on the surface as preferred adsorption configuration. Upon going to dense‐packed islands we however do not observe a clear correlation between the molecular assembly within the islands and the moiré superstructure of the substrate. Also the height variation of the molecules within the islands do not show a periodic modulation characteristic for the moiré structure, see Section S1, Supporting Information. We thus consider the substrate–molecule interaction to be substantially weaker than the molecule–molecule interaction for Dy_2_@C_80_(CH_2_Ph) SMMs on graphene/Ir(111).

**Figure 2:**
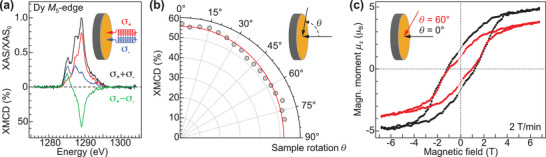
Magnetic properties of the Dy_2_@C_80_(CH_2_Ph) sub‐monolayer on graphene/Ir(111). a) XAS and XMCD spectra of Dy_2_@C_80_(CH_2_Ph) recorded at the Dy *M*_5_‐edge at normal incidence (θ = 0°). σ_−_ and σ_+_ refers to right and left circularly polarized light, respectively. Measurement parameters: *B* = 6.8 T, *T* = 2.5 K. b) Angular dependence of the absolute XMCD signal at 1289.0 eV ranging from θ = 0° (out‐of‐plane) to θ = 80° (grazing). The red line denotes a fit with the function XMCD ∝ cos^2^(θ), revealing a preferred orientation of the magnetic easy axes of a fraction of all molecules in out‐of‐plane direction. Measurement parameters: *B* = 6.8 T, *T* = 2.5 K. c) Magnetic hysteresis loops (0° and 60°) obtained by recording the XMCD signal at 1289.0 eV upon sweeping the magnetic field with 2 T min^−1^ at *T* = 2.5 K and a photon flux of 65.3Φ_0_. Φ_0_ = 1 x 10^−^^3^ photons nm^−^^2^s^−^^1^. The saturation values are expressed by the magnetic moments μ_z_ as obtained from sum rule analysis.

In order to explore the electronic and magnetic properties of the Dy_2_@C_80_(CH_2_Ph) SMM monolayer we employ synchrotron‐based XAS and XMCD techniques.^[^ ^35^ ^]^ **Figure** **2**a shows XAS spectra recorded at the Dy *M*_5_‐edge with both left (σ_+_) and right (σ_−_) circularly polarized light as well as the derived XMCD signal calculated as the difference (σ_+_ − σ_−_). All spectra are normalized to the maximum in the (σ_+_ + σ_−_) curve at 1289 eV (≡XAS_0_) and the XMCD signal is expressed in percent. The spectral shape indicates a Dy^3+^ (4f^9^) oxidation state^[^ ^36^, ^37^ ^]^ and coincides with measurements performed on related endohedral dimetallofullerenes.^[^ ^20^, ^27^, ^29^, ^38^ ^]^ The whole Dy *M*_4,5_ range spectra together with the corresponding sum rule analysis is presented in Section S2, Supporting Information. We deduce a magnetic moment of μ_z_ = 4.8 ± 1.1 μ_B_ per Dy^3+^ ion at normal incidence (θ = 0°). This reflects the previously observed scatter in the adsorption geometry of Dy_2_@C_80_(CH_2_Ph) on graphene/Ir(111) due to its reduced value compared to 10 μ_B_ per Dy^3+^ ion in the single molecule.^[^ ^28^, ^38^ ^]^

In order to obtain more detailed information about the orientation of the magnetic moments in the sample we track the XMCD signal upon changing the angle between beam incidence and surface normal from θ = 0° (out‐of‐plane) to θ = 80° (grazing), see Figure 2b and Figure S2b, Supporting Information. Whereas the spectral shape does not change we observe a variation of the relative XMCD intensity between 50–60%. The majority of Dy_2_@C_80_(CH_2_Ph) SMMs in the sub‐monolayer thus show no preferred orientation of the magnetic easy axis^[^ ^20^ ^]^ and only a fraction of molecules has their magnetic moments aligned in out‐of‐plane direction. The observed variation is also reflected as a slight reduction of the magnetic moment to μ_z_ = 3.5 ± 0.7 μ_B_ per Dy^3+^ ion at θ = 60° (see Figure S2b, Supporting Information). These findings show that in spite of the ordered assembly visible in STM only a minor effect toward a preferred alignment of the easy axis is present. This behavior is likely caused by the rotational degrees of freedom of both the fullerene body with respect to the addend as well as of the [Dy^3+^–*e*–Dy^3+^] spin center with respect to the C_80_ cage, both pointing at a weak molecule–substrate interaction.

We further acquire magnetization curves of the sample at normal (θ = 0°) and grazing (θ = 60°) incidence, which are presented in Figure 2c. At a magnetic field sweep rate of 2 T min^−1^ and *T* = 2.5 K we observe a hysteresis opening around ±4 T and a coercive field of about 1.2 T, comparable to values obtained by SQUID magnetometry at bulk samples.^[^ ^28^ ^]^ On the other hand we obtain a reduced ratio of remanent to saturation magnetization *M*_rem_/*M*_sat_ ≈ 30 % as compared to bulk, indicating a faster relaxation time for Dy_2_@C_80_(CH_2_Ph) in our experiment. The different saturation magnitude of both curves reflect the preferred orientation of Dy magnetic moments in out‐of‐plane direction.

**Figure 3:**
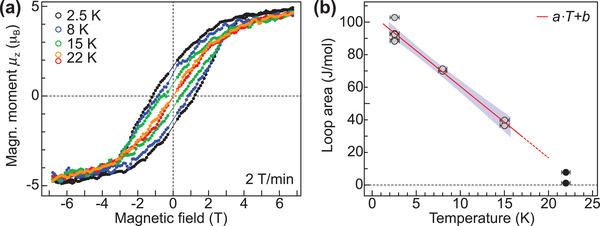
Magnetic hysteresis of the Dy_2_@C_80_(CH_2_Ph) sub‐monolayer on graphene/Ir(111). a) Magnetic hysteresis curves recorded at normal incidence (θ = 0°) and at different temperatures in the range of 2.5–22 K using a photon flux of 65.3Φ_0_. For the measurement at 22 K the variation of magnetization upon field sweeping is highlighted by different colors for both sweep directions (red = forward, orange = backward). b) Calculated hysteresis loop area as a function of the sample temperature. Multiple points per temperature denote different measurements of the magnetization curves. A linear fit up to 15 K yields *a* = −4.36 ± 0.30 J mol^−1^ K^−1^, *b* = 104.1 ± 2.5 J mol^−1^ and is plotted in red together with its 90% confidence interval as shaded area. The dashed red line highlights the extrapolation toward *T*_close_.

One of the key parameters in the characterization of surface‐supported molecular magnets is the closing temperature *T*_close_ of the magnetic hysteresis curve at a given magnetic field sweep rate. We therefore acquire magnetization curves at sample temperatures of 2.5, 8, 15, and 22 K in normal incidence and plot the results in **Figure** **3**a. The hysteresis loop gradually closes and suggests small but finite opening at 22 K. We calculate the loop area and obtain a linear trend with the temperature, see Figure 3b. Previous benchmark measurements on TbPc_2_ on a MgO insulating layer rather suggest an exponential decay,^[^ ^25^ ^]^ which is clearly not observed in our case. Even performing a defensive evaluation by omitting the point at 22 K due to a very small opening area, the linear fit in the range of 2.5−15 K (solid line in Figure 3b) suggests a closing temperature above 20 K, resembling the value obtained for the bulk.^[^ ^28^ ^]^ We note that in this case the fit perfectly reproduces the point at 22 K (dashed line in Figure 3b), suggesting its overall validity albeit measurement uncertainty. Our findings demonstrate the robust molecular magnetism of Dy_2_@C_80_(CH_2_Ph) on graphene/Ir(111) that sustains up to record‐high temperatures at about 20 K for surface‐supported molecular magnets.

**Figure 4:**
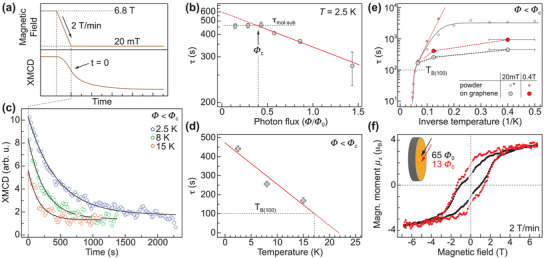
Magnetic relaxation of the Dy_2_@C_80_(CH_2_Ph) sub‐monolayer on graphene/Ir(111). a) Measurement scheme of the experiment as explained in the text. b) Magnetic relaxation time τ as function of X‐ray photon flux in normal incidence and 2.5 K. A horizontal dashed line marks the average value in the regime Φ < Φ_c_, a fit (red) denotes photon‐induced demagnetization as dominant process for Φ > Φ_c_. For the vertical axis we use a reciprocal scale. c) Time traces of the XMCD signal recorded at 2.5 K (blue), 8 K (green), and 15 K (orange). Exponential fits with a function XMCD ∝ exp(−*t*/τ) + XMCD_0_ are presented as solid lines. d) Temperature‐dependency of the relaxation time τ at 20 mT. As lowest order approximation a linear fit is sketched as solid red line, the determination of *T*_B(100)_ is marked. e) Magnetic‐field dependency of the relaxation time τ. Diamonds and solid curves denote data obtained by SQUID on a bulk sample (data marked by asterisk is for 0 T) and corresponding fits, respectively.^[^ ^28^ ^]^ Dashed lines serve as guide to the eye. Error bars for τ are smaller than the size of data points and thus not shown. f) Magnetic hysteresis recorded at a photon flux of 65Φ_0_ (black) and 13Φ_0_ (red). The curves are acquired at grazing incidence in order to maximize the signal‐to‐noise ratio. Measurement parameters: θ = 60°, *T* = 2.5 K.

We now focus on the magnetization relaxation dynamics of the Dy_2_@C_80_(CH_2_Ph) sub‐monolayer. In order to disentangle the contribution of X‐ray photon‐flux‐induced and pure adsorption‐related demagnetization we perform time‐dependent XMCD relaxation measurements. The results are plotted in **Figure** **4**a–e and Figure S3, Supporting Information. The XMCD signal was recorded as a function of time at *B*_rest_ = 20 mT after saturating the magnetization at 6.8 T. In all measurements we observe a behavior that can be fitted with an exponential decay XMCD ∝ exp(−*t*/τ) + XMCD_0_. Whereas XMCD_0_ depends on the thermodynamic equilibrium at *B*_rest_, the relaxation time τ provides a measure for the internal demagnetization dynamics of Dy_2_@C_80_(CH_2_Ph) on graphene/Ir(111).^[^ ^4^, ^18^, ^25^ ^]^ We note that small oscillations visible in the XMCD time traces are related to signal intensity fluctuations due to the top‐up operation mode of the synchrotron.

Figure 4b shows the magnetic relaxation time τ as a function of X‐ray photon flux Φ under normal incidence. We observe a clear flux‐induced decrease of the relaxation time for values larger than Φ_c_ ≈ 0.4Φ_0_, with Φ_0_ = 1 × 10^−3^ photons nm^−^^2^ s^−1^. The dominant contribution of only one relaxation channel in this regime allows us to deduce the X‐ray demagnetization cross‐section (see Section S3, Supporting Information). The significant decrease explains the smooth shape of the hysteresis curves in Figures 2c and 3a, both recorded using a photon flux of Φ_large_ = 65.3Φ_0_ (see Section 2). The striking feature in the measurement however is a clear plateau at X‐ray intensities below Φ_c_ where no change of the relaxation time is observed, marked as dashed line at τ_mol‐sub_ = 463 ± 11 s. We explain this saturation by a crossover to a time scale that is dominated by intrinsic demagnetization processes. To minimize the influence of X‐ray induced demagnetization we thus used a photon flux of 0.28 Φ_0_ < Φ_c_ for the following experiments in order to analyze the magnetization dynamics of the system.

As the closing temperature of the hysteresis loop is just a rough estimate of the blocking temperature of a molecular magnet and highly depends on the measurement parameters, *T*_B(100)_ has been established as reliable characteristics in the SMM community. It denotes the temperature at which the relaxation time of a system amounts to 100 s, independently of the involved relaxation processes. In Figure 4c,d we plot XMCD time traces and the extracted values of τ as a function of sample temperature. In lowest order approximation the data can be fitted with a linear relation which yields *T*_τ=0s_ = 21.7 ± 6.9 K, being in good agreement with the value estimated from temperature dependence of the hysteresis loop area. This allows us to infer a magnetic blocking temperature of *T*_B(100)_ = $17.1_{-2.1}^{+5.7}$ K, which matches the bulk value.^[^ ^28^ ^]^ Whereas the positive boundary denotes the standard error of the linear fit, the lower boundary is the difference to the 15 K measurement at which the relaxation time is still considerably larger.

In order to corroborate our experimental results we compare the surface‐induced relaxation to bulk behavior studied by SQUID magnetometry,^[^ ^28^ ^]^ see Figure 4e. Whereas τ resembles the bulk value at 15 K, it is strongly reduced compared to τ_bulk_ at lower temperatures. We assign this suppression to both X‐ray induced demagnetization that limits the relaxation time as discussed before as well as to zero‐field quantum tunneling of magnetization (QTM) observed in bulk,^[^ ^28^ ^]^ which can be modified upon surface adsorption.^[^ ^21^ ^]^ To further evaluate on this, we apply a magnetic field of 0.4 T, leading to an increase of τ to 941 ± 86 s at 2.5 K for the surface adsorbed SMM. We note that this value represents a lower margin for the magnetic relaxation time due to the present X‐ray induced demagnetization, which remains unaffected by the magnetic field. The increase of τ in a magnetic field points to a suppression of QTM upon lifting the degeneracy of the spin ground states. At the same time it excludes an increased coupling to phonon modes that should become available with larger splitting.^[^ ^39^ ^]^ Thus a takeover of Raman scattering as observed in bulk is likely to be the main driving mechanism of magnetic relaxation, additionally enhanced by the involvement of substrate phonons. In particular, phonon‐induced relaxation pathways upon coupling to acoustic (ZA) phonons of graphene around 58 meV,^[^ ^40^, ^41^ ^]^ roughly corresponding to the effective barrier height of 615 K for Dy_2_C_80_(CH_2_Ph), should be considered. Moreover, the non‐negligible coupling to the conduction electrons could open an additional relaxation channel involving spin‐flip scattering.^[^ ^21^ ^]^ However, a more detailed investigation is inevitable to address these points. Apart from these considerations for low temperatures, the exceptional concordance with bulk relaxation dynamics at 15 K as suggested from hysteresis and relaxation measurements supports the bulk‐like value determined for *T*_B(100)_. This furthermore points to a relaxation at 15 K that is likely dominated by an unusual thermally activated Orbach process with low barrier as observed in the bulk,^[^ ^28^ ^]^ where the graphene surface plays no dominant role anymore. Conventional Orbach relaxation involving the first excited spin states is expected to prevail at even higher temperatures, which is however not assessed in these measurements.

We now record the magnetization curve using the lowest feasible photon flux of Φ_low_ = 0.2Φ_large_ = 13.1Φ_0_ (see Section 4 for further details). We note that the used X‐ray flux still exceeds Φ_c_ by far, but turned out to be the best compromise between viable signal‐to‐noise ratio and total measurement time. The hysteresis loops of both photon fluxes for an angle of θ = 60° are shown in Figure 4f. A strongly enhanced relaxation time leads to a further substantial opening of the hysteresis curve. First, this implies an increase of the coercive field by roughly +25% to 1.3 T. Second, despite a waiting time of 20 s at zero field it furthermore leads to an increase of the magnetic remanence ratio *M*_rem_/*M*_sat_ to roughly 50%, representing the largest value found for SMMs adsorbed on surfaces.^[^ ^25^ ^]^ The substantial opening of the hysteresis curve at low X‐ray flux as well as the remarkable agreement of the estimated blocking temperature *T*_B(100)_ with the bulk value suggests only very weak impact of the graphene substrate on the [Dy^3+^–*e*–Dy^3+^] molecular core and its ligand field. Starting with the first observation of slow magnetic relaxation in surface‐supported SMMs over 10 years ago in the sub‐Kelvin regime^[^ ^11^ ^]^ and subsequent improvements towards 10 K,^[^ ^24^, ^25^, ^26^, ^27^ ^]^ this work demonstrates a significant increase of the on‐surface blocking temperature compared to all previous studies. Furthermore, our system is excellently suited to address the spin states and magnetization dynamics at a single‐molecule level, for example using local probe methods.^[^ ^42^, ^43^ ^]^

## 3 Conclusion

We have demonstrated that the Dy_2_@C_80_(CH_2_Ph) SMM widely retains its magnetic properties upon surface deposition. This is achieved by a combination of the electrospray technique as gentle deposition method together with the use of graphene/Ir(111) as an appropriate substrate. The sub‐monolayer shows a hysteresis curve that closes around 20 K, twice the value measured on surface‐adsorbed molecular magnets so far. We disentangle the contribution of the X‐ray photon flux on the demagnetization dynamics by employing time‐dependent XMCD relaxation measurements and obtain a magnetic blocking temperature of *T*_B(100)_ ≈ 17 K. This represents the highest value for surface‐supported molecular magnets reported up to date and most notably matches at the same time the bulk blocking temperature. Our study thus provides a new milestone on the path toward on‐surface systems with application‐relevant working temperatures. Moreover, it demonstrates that the graphene–dimetallofullerene system represents a suitable platform for future studies of complex spin systems at the scale of single molecules, aiming at advanced information processing and memory units.

## 4 Experimental Section

All samples were prepared in situ. The Ir(111) single crystal (Surface Preparation Laboratory B. V.) was cleaned by repeated cycles of Ar^+^ sputtering at 2 kV, heating in an O_2_ atmosphere of 5 × 10^−7^ mbar at 900–1150 °C and flash annealing in ultra‐high vacuum (UHV) up to 1500 °C. Graphene was prepared by exposing the clean Ir(111) surface to an ethylene atmosphere at a pressure of 1.1 × 10^−7^ mbar for 20 min while keeping the sample at *T* = 1200°C. Dy_2_@C_80_(CH_2_Ph) molecules were freshly solved in 1,2‐dichlorobenzene and deposited in situ by ESD while the sample was kept at room temperature. The used ESD setup is described in detail elsewhere.^[^ ^34^ ^]^ In the presented XAS/XMCD study a total amount of 0.11 monolayer of Dy_2_@C_80_(CH_2_Ph) has been deposited on graphene/Ir(111) to obtain a sub‐monolayer coverage. The samples were prepared in situ using the very same procedure both at the low‐temperature STM home lab and at the beamline in order to ensure comparable coverage and sample quality.

Scanning tunneling microscopy and spectroscopy experiments were performed in a two‐chamber UHV system (base pressure 5 × 10^−11^ mbar), equipped with an Omicron Cryogenic‐STM. All STM measurements were carried out in the constant‐current mode using grinded and polished PtIr tips (Nanoscore GmbH). The sign of the bias voltage (*U*) corresponds to the potential applied to the sample.

XAS and XMCD experiments were performed at the X‐Treme beamline^[^ ^44^ ^]^ of the Swiss Light Source at the Paul Scherrer Institute. The measurements were performed in the total electron yield mode, with the magnetic field being aligned antiparallel to the incoming X‐ray beam and forming an angle θ with the surface normal of the sample. Special care had been taken in order to center the beam by rechecking the sample position and signal every multiple of 10°. At θ = 80°, the projected beam spot width on the surface is 2.8 mm, being still considerably smaller than the sample width of 4 mm. The measurements were performed using a defocused X‐ray beam in order to minimize the photon flux. Accordingly, no significant beam damage could be observed in the spectra throughout the measurements. For magnetization curves no data points were collected within *B* = ±0.2 T at a magnetic field sweep rate of 2 T min^−1^ due to polarization reversal of the coil current, which also implied a waiting interval of around 20 s at zero field. XMCD relaxation measurements were performed by continuously alternating between both polarizations σ_+_ and σ_−_. The resulting curve was then calculated by interpolation and subtraction of both curves, XMCD(*t*) = σ_+_(*t*) − σ_−_(*t*). In order to minimize the photon flux the beam shutter was only opened for *t*_acqu_ = 0.5 s with a waiting time of *t*_reversal_ = 22.3 s in between each point due to polarization reversal. The flux for the relaxation measurements thus had been corrected using $\phi \;=\;\phi_{\text{cw}}\frac{t_{\text{acqu}}}{t_{\text{acqu}}\;+\;t_{\text{reversal}}}$,^[^ ^4^ ^]^ with Φ_cw_ being the respective continuous flux value depending on the beam spot size.^[^ ^44^, ^45^ ^]^

## Conflict of Interest

The authors declare no conflict of interest.

## Author Contributions

F.L. and A.A.P. synthesized the Dy_2_@C_80_(CH_2_Ph) molecules. F.P. and T.B. performed surface deposition and STM characterization. F.P., T.B., V.E., M.F., V.R., and J.D. performed the XAS/XMCD experiments. M.F. conceived and supervised the experiments. F.P. analyzed STM and XMCD data. F.P. and M.F. drafted the manuscript. All authors discussed the results and contributed to writing the paper.

## Supporting information

Supporting Information

## Data Availability

The data that support the findings of this study are available from the corresponding author upon reasonable request.
