# Supplementary material for: Exceptionally High Blocking Temperature of 17 K in a Surface‐Supported Molecular Magnet
Source: Adv Mater. 2021 Aug 15;33(40):2102844. doi: 10.1002/adma.202102844 (PMC11468252; doi:10.1002/adma.202102844)
Supplement: Supplementary file 1 — Supporting Information [file ADMA-33-2102844-s001.pdf]

© 2021 Wiley-VCH GmbH

ADVANCED MATERIALS

Supporting Information

for *Adv. Mater.,* DOI: 10.1002/adma.202102844

Exceptionally High Blocking Temperature of 17 K in a Surface-Supported Molecular Magnet

Fabian Paschke, Tobias Birk, Vivien Enenkel, Fupin Liu, Vladyslav Romankov, Jan Dreiser, Alexey A. Popov, and Mikhail Fonin**

Supporting Information

Exceptionally high blocking temperature of 17 K in a surface-supported molecular magnet

Fabian Paschke,*,† Tobias Birk,† Vivien Enenkel,† Fupin Liu,‡ Vladyslav Romankov,¶ Jan Dreiser,¶ Alexey A. Popov,‡ and Mikhail Fonin*,†

†*Department of Physics, University of Konstanz, 78457 Konstanz, Germany*

‡*Leibniz Institute for Solid State and Materials Research (IFW Dresden), 01069 Dresden, Germany*

¶*Swiss Light Source, Paul Scherrer Institute, 5232 Villigen, Switzerland*

E-mail: fabian.paschke@uni-konstanz.de; mikhail.fonin@uni-konstanz.de

S1. STM data analysis

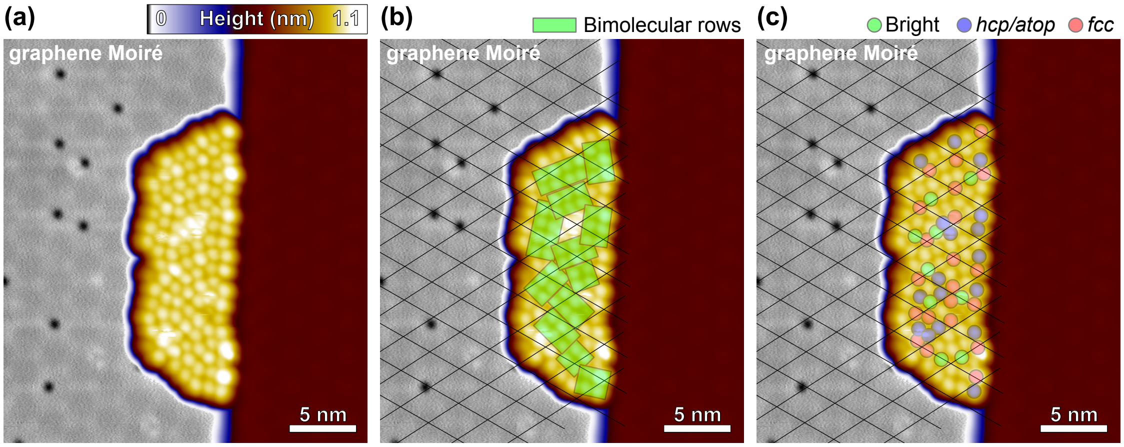

Figure 1: **STM analysis of a $Dy_2@C_{80}(CH_2Ph)$ submonolayer on graphene/Ir(111).**

The ordering of $Dy_2@C_{80}(CH_2Ph)$ molecules on the graphene Moiré can be examined in Fig. S1a, which displays the same island as shown in Fig. 1b of the main text. The color scale is adjusted to highlight both the graphene Moiré superlattice as well as the surface of the molecular island. To reveal possible correlations between surface structure and molecular ordering, a lattice corresponding to the Moiré structure is superposed on the STM image in Fig. S1b and c as black lines, with the crossings denoting the *fcc* lattice sites as in the main text. The molecules tend to form a densely packed hexagonal lattice, which is hindered by the CH_2Ph side group that leads to local formation of tetragonal arrangement in bimolecular rows, marked as green rectangles in Fig. S1b. No preferred alignment of molecular rows along the graphene high symmetry directions can be observed. Furthermore, Fig. S1c shows all molecules that appear slightly brighter than their neighbors. Those which are sitting near the *fcc* lattice sites are marked in red ($N = 21$), those near the *hcp/atop* lattice sites in blue ($N = 17$) and all others in green ($N = 9$). We therefore observe no particular correlation between bright molecules and the underlying Moiré superlattice as it is the case for other molecules on comparable substrates.[1] The different appearance is thus likely related to different adsorption orientations within the molecular islands, as described in the main text.

S2. XAS/XMCD and sum rule analysis

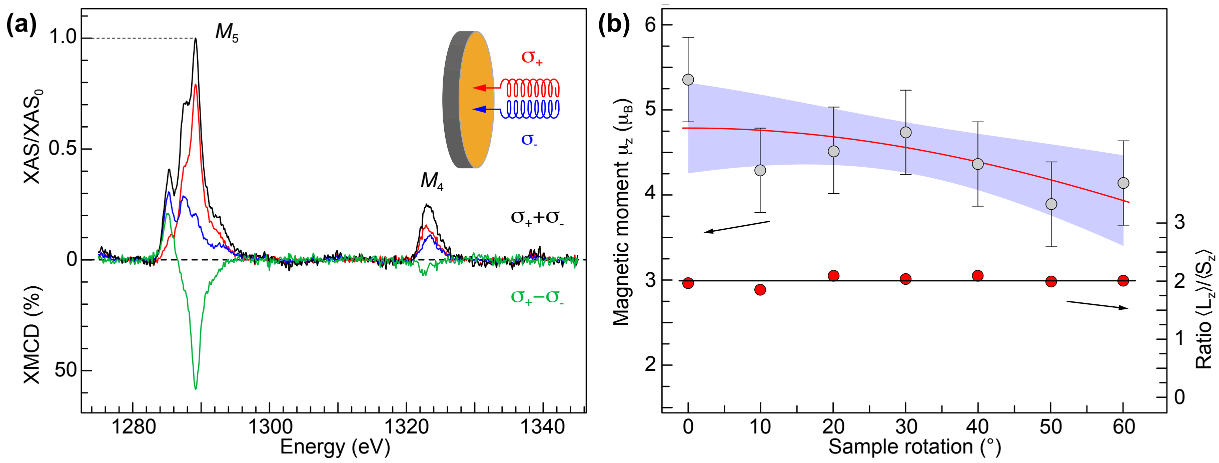

Figure 2: **XAS/XMCD analysis of $Dy_2@C_{80}(CH_2Ph)$ submonolayers on graphene/Ir(111).**

Raw XAS spectra were background-corrected by fitting a polynom of sufficiently high order to each spectrum under exclusion of the energy ranges around the Dy $M_{4,5}$ absorption edges (1282 − 1296 eV and 1318 − 1330 eV). The XAS spectra are then calculated using XAS = $\frac{\mathrm{XAS_{raw}}}{\mathrm{XAS_{background}}}$ and normalized by division to the maximum of $(\sigma_+ + \sigma_-)$ at 1289 eV, named XAS_0. The obtained typical XAS spectra and the corresponding XMCD signal measured under normal incidence and at 2.5 K are shown in Figure S2a. We employ sum rule analysis as developed for 4f-elements to calculate the expectation values of the spin angular momentum operator $\langle \hat{S}_z \rangle$ and orbital angular momentum operator $\langle \hat{L}_z \rangle$ in units of $\hbar$ by using[2,3]

$$\langle \hat{L}_\mathrm{z} \rangle = 3\,(14 - n_\mathrm{4f})\,\frac{\int_{M4+M5}(\sigma_+ - \sigma_-)\mathrm{d}\omega}{\int_{M4+M5}(\sigma_+ + \sigma_- + \sigma_0)\mathrm{d}\omega} \quad (1)$$

$$2\,\langle \hat{S}_\mathrm{z} \rangle + 6\,\langle \hat{T}_\mathrm{z} \rangle = C_{\mathrm{Dy}3+} \cdot 3\,(14 - n_\mathrm{4f})\,\frac{\int_{M5}(\sigma_+ - \sigma_-)\mathrm{d}\omega - \frac{3}{2}\int_{M4}(\sigma_+ - \sigma_-)\mathrm{d}\omega}{\int_{M4+M5}(\sigma_+ + \sigma_- + \sigma_0)\mathrm{d}\omega} \quad (2)$$

$n_\mathrm{4f} = 9$ denotes the number of electrons in the Dy^{3+} 4f shell and $M_{4,5}$ are the respective XAS absorption edges for integration. The isotropic XMCD signal is expressed as $\sigma_0 = \frac{1}{2}(\sigma_+ + \sigma_-)$ as commonly reported in the literature[4,5] as a good estimation for systems with

small magnetic anisotropy as in the case of $Dy_2@C_{80}(CH_2Ph)$ on graphene/Ir(111). Thus the denominators in equations 1 and 2 read $\frac{3}{2}B$ with $B = \int_{M4+M5}(\sigma_+ + \sigma_-)$ being the area covered by the black curve in Figure S2a. $C_{\mathrm{Dy3+}}$ denotes a correction factor as determined for trivalent rare earth elements and has a value of 1.088 for Dy^{3+}.[3] Furthermore one has to take into account the spin dipole operator $\hat{T}_\mathrm{z}$ in order to deduce the expectation value $\langle \hat{S}_\mathrm{z} \rangle$. We use a ratio of $\frac{\langle \hat{T}_\mathrm{z} \rangle}{\langle \hat{S}_\mathrm{z} \rangle} = -0.053$ as determined by atomic calculations of Dy^{3+}.[3] The magnetic moments are then calculated using $\mu_\mathrm{z} = \frac{\mu_\mathrm{B}}{\hbar}\left(\langle \hat{L}_\mathrm{z} \rangle + 2\langle \hat{S}_\mathrm{z} \rangle\right)$.

Sum rule analysis on a series of XAS spectra at the Dy $M_{4,5}$ edges that were recorded upon variation of the angle between beam incidence and surface normal yields only small angular dependency of magnetic moments of the submonolayer. The magnetic moments μ_z as derived from equations 1 − 2 are plotted in Figure S2b. Large error bars of $\pm 0.5\,\mu_\mathrm{B}$ per Dy atom denote the uncertainty of sum rule analysis concerning correct background-removal and integration borders. We observe a slight decrease of the magnetic moment when approaching grazing incidence around $\theta \approx 60°$ which resembles the evolution of the XMCD signal at 1289.0 eV as plotted in Figure 2b in the main text. Therefore the data can be fitted with a $\propto \cos^2(\theta)$ behavior, plotted as solid black line with its 90 % confidence interval in light blue. This leads to $\mu_{\mathrm{z},0°} = 4.79 \pm 1.09\,\mu_\mathrm{B}$ per Dy^{3+} ion at normal incidence and $\mu_{\mathrm{z},60°} = 3.51 \pm 0.73\,\mu_\mathrm{B}$ per Dy^{3+} ion at grazing incidence. These values are in good agreement with previous XMCD studies on related endohedral metallofullerenes and reflect a slightly preferred magnetic easy axis pointing in the out-of-plane direction.[6] In Figure S2b we additionally plot the ratio between orbital and spin angular momentum $\langle \hat{L}_\mathrm{z} \rangle / \langle \hat{S}_\mathrm{z} \rangle$ as obtained from sum rule analysis. The values fluctuate around a mean value of 1.999 ± 0.033 (solid red line), perfectly matching the value of 2 as expected for Dy^{3+} ions.[3] To our knowledge, no ratios have been reported in the literature for lanthanide-based molecular magnets comparable to $Dy_2@C_{80}(CH_2Ph)$ so far.

S3. Flux-dependent XMCD relaxation measurements

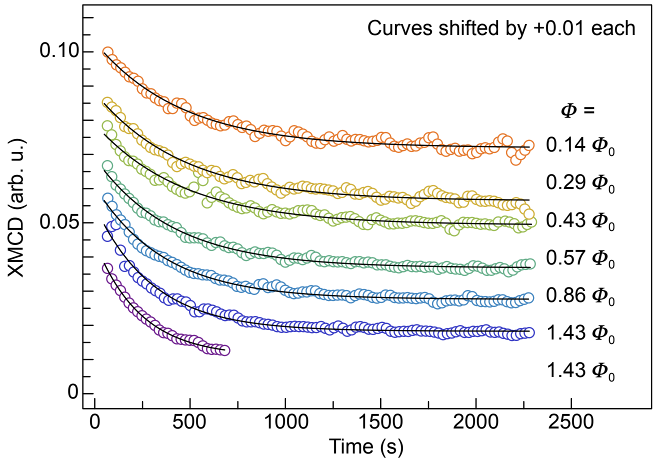

Figure 3: **X-ray photon flux dependency of XMCD relaxation of $Dy_2@C_{80}(CH_2Ph)$ submonolayers on graphene/Ir(111).**

XMCD time traces recorded for different X-ray photon fluxes are displayed in Figure S3, for clarity the curves are vertically shifted by $\Delta_i = 0.01 \cdot i$. The corresponding fitted relaxation times τ are shown in Figure 4b in the main text. For the regime $\Phi > 0.4\Phi_0$, where X-ray induced demagnetization appears to be the dominant mechanism, the total relaxation time can be expressed as combination of different relaxation channels, which in a low order approximation reads[7,8]

$$\tau^{-1}(\Phi) = \tau^{-1}_{\Phi=0} + \sigma\Phi \tag{3}$$

with $\tau_{\Phi=0}$ as intrinsic relaxation time-scale at zero flux and σ denoting the X-ray demagnetization cross-section. We use this expression to fit our data in the range of $\Phi > 0.4\Phi_0$ and obtain $\tau_{\Phi=0} = 601 \pm 51\,\mathrm{s}$ and $\sigma = 12.9 \pm 2.1\,\mathrm{Gb}$. The resulting curve is plotted as straight line in Figure 4b in the main text. Whereas the first quantity is among the longest relaxation times reported for surface-adsorbed single molecular magnets as pointed out in the main text, σ lies in the same order of magnitude of values obtained on comparable SMM submonolayers on surfaces due to resonant excitation at the M_5-edge.[8,9]

References

(1) A. Kumar, K. Banerjee, P. Liljeroth, Nanotech. **28**, 082001 (2017).

(2) B. T. Thole, P. Carra, F. Sette, G. van der Laan, Phys. Rev. Lett. **68**, 12 (1992).

(3) Y. Teramura, A. Tanaka, B. T. Thole, T. Jo, J. Phys. Soc. Jpn. **65**, 9 (1996).

(4) C. T. Chen, Y. U. Idzerda, H.-J. Lin, N. V. Smith, G. Meigs, E. Chaban, G. H. Ho, E. Pellegrin, F. Sette, Phys. Rev. Lett. **75**, 1 (1995).

(5) A. Singha, R. Baltic, F. Donati, C. Wäckerlin, J. Dreiser, L. Persichetti, S. Stepanow, P. Gambardella, S. Rusponi, H. Brune, Phys. Rev. B **96**, 224418 (2017).

(6) D. S. Krylov, S. Schimmel, V. Dubrovin, F. Liu, T. T. N. Nguyen, L. Spree, C.-H. Chen, G. Velkos, C. Bulbucan, R. Westerström, M. Studniarek, J. Dreiser, C. Hess, B. Büchner, S. M. Avdoshenko, A. A. Popov, Angew. Chem. Int. Ed. **59**, 5756–5764 (2020).

(7) F. Donati, S. Rusponi, S. Stepanow, C. Wäckerlin, A. Singha, L. Persichetti, R. Baltic, K. Diller, F. Patthey, E. Fernandez, J. Dreiser, Ž. Šljivančanin, K. Kummer, C. Nistor, P. Gambardella, H. Brune, Science **352**, 6283 (2016).

(8) J. Dreiser, R. Westerström, C. Piamonteze, F. Nolting, S. Rusponi, H. Brune, S. Yang, A. Popov, L. Dunsch, T. Greber, Appl. Phys. Lett. **105**, 032411 (2014).

(9) C. Wäckerlin, F. Donati, A. Singha, R. Baltic, S. Rusponi, K. Diller, F. Patthey, M. Pivetta, Y. Lan, S. Klyatskaya, M. Ruben, H. Brune, J. Dreiser, Adv. Mater. **28**, 5195–5199 (2016).
